# Usage of Meropenem Continuous Infusion for Treatment of Infectious Complications in Orthopedic Elderly Patients with Anemia: A Case Series

**DOI:** 10.3390/medicina60060929

**Published:** 2024-06-02

**Authors:** Aidos Konkayev, Assiya Kadralinova, Benazir Azimova, Damira Tazhibayeva, Aigerim Yeltayeva, Maiya Konkayeva

**Affiliations:** 1 Department of Anesthesiology and Intensive Care, National Scientific Center of Traumatology and Orthopedics Named after Academician N.D. Batpenov, Astana 010000, Kazakhstan; ysaimnida@mail.ru (A.K.); al_paka@mail.ru (B.A.); 8602817@mail.ru (A.Y.); mkonkaeva@mail.ru (M.K.); 2 Department of Anesthesiology and Intensive Care, Astana Medical University, Astana 010000, Kazakhstan; tazhybayeva.d@amu.kz

**Keywords:** infection, meropenem continuous infusion, orthopedic complications, anemia, case report

## Abstract

*Background and Objectives:* The prolonged infusion of meropenem is recommended by guidelines for the treatment of sepsis. However, studies provide controversial data on the advantages of prolonged infusions over intermittent ones. In our opinion, this can be related to age, which possibly distorts the final data, as older people have age-related characteristics. In our study, we analyzed the ventilatory status, laboratory tests and vital signs of the patient and carried out microbiological cultures. *Materials and Methods:* This was a prospective single-center case series investigation conducted from June 2022 to June 2023. The objective of this study was to evaluate the effectiveness of continuous infusion in elderly patients with severe infectious complications after orthopedic interventions. The primary endpoints were 28-day survival and the emergence of new multidrug-resistant strains. Secondary endpoints were long-term mortality and length of stay in the ICU. *Results*: Three patients (median age 65, 100% female) received a continuous infusion of meropenem. Two patients were alive at hospital discharge, and one patient died on the 105th day of hospitalization. Multi-resistant bacteria were observed in one patient. *Conclusions:* The use of a continuous meropenem infusion in the complex treatment of purulent-septic complications in elderly patients with periprosthetic infection and anemia probably led to clinical improvement in these case reports. However, the emergence of new pan-resistant strains and overall mortality using this infusion technique remains unclear. Further, high-quality RCTs for the elderly are needed.

## 1. Introduction

The use of the continuous infusion of carbapenems in severe infections, including sepsis, is widely studied all over the world. In particular, many studies have been conducted on the use of prolonged meropenem infusion in pediatric practice. Also, a large number of studies are being conducted separately on the topic of the prolonged infusion of meropenem in adults. Most studies show the greatest effectiveness of using extended meropenem infusion, but there are studies in which there are no differences in effectiveness between bolus and prolonged meropenem infusion, depending on the expected results. In turn, categories of geriatric patients, as well as children, should be considered separately due to the presence of age-related features in drug metabolism.

According to the World Health Organization, by 2050, it is expected that the proportion of the world’s inhabitants over 60 years of age will double and reach 22% [1]. Since aging is accompanied by changes in immune response, this will lead to an increase in the growth of age-related diseases due to the increased susceptibility of the body to infections, cancer, and cardiovascular and neurodegenerative diseases. Infections lead to an unregulated immune response and, consequently, the dysfunction of vital organs. Worldwide, sepsis affects 49 million people and leads to 11 million sepsis-related deaths [2]. According to the study by Palomba H. et al., age was found to be an independent predictor of death in patients with sepsis and septic shock [3]. This phenomenon is probably associated with the peculiarities of the immune response in the elderly, which is a condition called immunosenescence. All over the world, studies are conducted on adults, starting at 18 years of age. In our opinion, this possibly distorts the final data since older people have their own age characteristics. And, therefore, isolated studies are needed that are applicable to the elderly. In particular, this applies to antibiotic therapy in the treatment of sepsis. The new Surviving Sepsis Campaign Guideline recommends the use of prolonged infusions of meropenem in sepsis to maintain a therapeutic dose of the antibiotic in the blood [4]. Due to the characteristics of immune response in the elderly, as well as their slow metabolism and reduced kidney function, this approach to antibiotic therapy may be of particular concern. This article highlights three clinical cases of continuous meropenem infusion for sepsis in orthopedic elderly patients.

## 2. Materials and Methods

This was a prospective single-center case series investigation conducted from June 2022 to June 2023 at the National Scientific Center of Traumatology and Orthopedics, named after academician Batpenov N.D., in Astana, Kazakhstan, and was approved by the Local Ethics Committee of our institution.

Inclusion criteria were patients over 60 years of age with a diagnosis of sepsis (q-SOFA scale over two points) and the presence of periprosthetic infection and concomitant anemia. The study population was a convenient sample of patients because sepsis was associated with periprosthetic infection. Exclusion criteria were having an allergy or individual intolerance to the study drug, unfavorable prognosis by the SAPS II scale (over 65 points), and individuals with a concomitant acquired immunodeficiency syndrome (stage 3, according to the CDC).

Meeting the inclusion and exclusion criteria, respondents were invited to participate in the study. After informed consent was obtained, the study patients received a continuous 24 h infusion of meropenem (Santo, Shymkent, Kazakhstan). Meropenem at a dose of 500 mg was diluted in 50 mL of NaCl 0.9% (10 mg/mL) and administered at a rate of 12.5 mL/h. A dosage adjustment was made considering creatinine clearance. The stability of the prepared antibiotic solution was ensured by preparing a completely new solution of the drug every 4 h. Such a methodic infusion was recommended and described in the MERCY study protocol [5].

Further, daily from the first to the eighth day of the study, ventilatory status (ventilated or not, invasive ventilation or not), vital signs, an arterial blood gas sample, serum electrolytes, total protein, C-reactive protein, complete blood count, clotting tests, liver (bilirubin, AST, ALT) and kidney (creatinine, urea) profiles, urine output, Glasgow coma scale evaluation and inotropic support were obtained.

Microbiological samples (blood, respiratory samples and urine) were collected before starting meropenem therapy 48 h later on days 3, 8 and 28 of the study.

The main result of this study is a descriptive analysis of the in-hospital mortality of elderly patients, as well as an analysis of microbiological crops and the appearance of new multi-resistant bacteria. Secondary outcomes included overall mortality, number of days in intensive care and number of days in hospital.

Descriptive statistics were used to report study outcome data. Data were collected in password-protected software and analyzed using commercial software (Microsoft Excel V16.43 20110804).

## 3. Case Presentation

### 3.1. Case 1

A 66-year-old woman was admitted to the hospital for the treatment of a periprosthetic joint infection with complaints of pain, limited movement and impaired function of the left hip joint. According to the patient’s medical history, in February 2021, she suffered a hip fracture as a result of a fall. She received conservative treatment and immobilization with a derotational boot for 4 months, but a false femoral joint was formed. Consequently, in September 2021, she underwent endoprosthetic surgery with a Stryker bipolar implant. Further, she noted the formation of fistulous wounds along the postoperative scar of the left hip joint, with purulent discharge. In December 2021, she underwent revision endoprosthesis with an antibiotic-loaded cement spacer. Nevertheless, in May 2022, she received in-patient non-surgical treatment for periprosthetic joint infection with temporary success.

The patient’s background was complicated by the presence of chronic iron deficiency anemia, hypertension and chronic cerebral ischemia of mixed genesis. Since 2018 she had been disabled due to left-sided spastic hemiplegia after suffering an ischaemic stroke.

**Figure 1:**
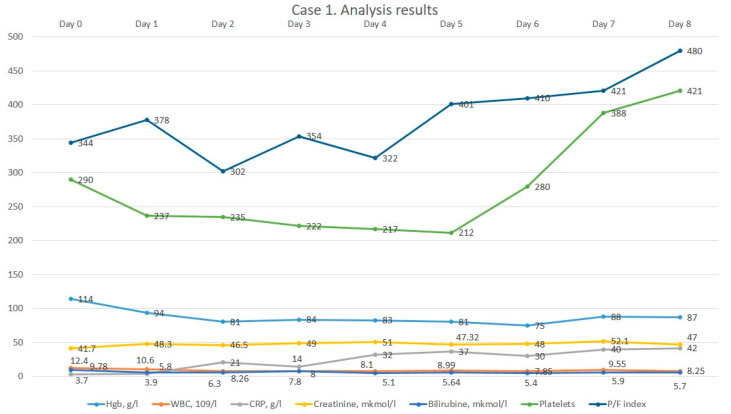
The dynamics of the 1st patient’s analysis results.

On initial examination, the patient lay in a forced position, passive. The patient’s temperature, respiratory and heart rate were normal. She was hypertonic with blood pressure 140/70 mmHg. The left lower extremity was shortened and rotated outwards and the symptom of “stuck heel” was positive. The left hip joint was deformed according to the “halife” type, and movements were severely limited and painful. There was a postoperative scar on the external surface of the left hip joint without peculiarities. Daily activity, according to the Barthel index, was 60 points, and according to Karnovsky, 40 points. The MRS-scale BMB was one point. The initial laboratory tests revealed no pathology other than mild anemia. Instrumental data revealed chronic bronchitis on X-ray and signs of reflux-gastritis on fibrogastroduodenoscopy; an ultrasound of the heart and vessels showed no abnormalities. The patient received 1 g of ceftriaxone two times a day for a week empirically, antihypertensive drugs situationally and analgesics lornoxicam at 8 mg intravenously and ketoprofen 200 mg intramuscularly. On the 7th day of hospital stay, she underwent surgical treatment for the PJI removal of a non-articulating spacer of the left hip joint, arthrotomy and sanation under spinal anesthesia with a blood loss of 300 mL. After the surgery, she was admitted to the ICU for postoperative care. The diagnosis of sepsis was based on the presence of infection and an SOFA score of two points. After starting continuous therapy with meropenem, moderate leucocytosis decreased to normal values. However, CRP dynamics rose, most likely as a result of surgical intervention. The dynamics of the patient’s laboratory parameters are shown in Figure 1.

As can be seen on the graph, the patient initially had a relatively low oxygenation index despite the oxygen therapy, after which the patient’s respiratory parameters gradually improved.

The patient stayed nine days in the intensive care unit in total; after her condition improved, she was moved to a specialized department for further therapy. By the time of discharge, the patient’s motor function had improved: her Bartel index score increased to 80, and Karnovsky index increased to 60.

### 3.2. Case 2

A 65-year-old female patient was admitted to the hospital complaining of localized oedema, hyperemia of tissues, pain in the left hip joint, and a rise in body temperature to 37.4 °C. Previously, the patient had undergone a total left hip replacement in December 2006. The patient experienced a recurrence of the pain syndrome a few months later, which led to surgery in June 2013 to replace the acetabular component of their left hip joint endoprosthesis. However, in January 2021, a re-prosthetic left hip replacement utilizing an acetabulum and Muller strengthening ring was carried out due to the appearance of pain syndrome. Further, in June 2022, due to the deterioration of the patient’s condition and the recurrence of the pain syndrome, she was admitted to the hospital for surgical treatment. The patient’s history included chronic iron deficiency anemia, atrial fibrillation, and arterial hypertension. She took 5 mg of bisoprolole on occasion. From previous surgeries, she also underwent appendectomy and the removal of an echinococcal cyst of the liver. Allergological anamnesis was not complicated.

The patient’s general condition on admission was relatively stable. The skin and visible mucous membranes were both the normal color, and the body temperature was 37.0 °C. A clean, rhythmic heartbeat with an arterial pressure of 130/80 mm Hg and a heart rate of 76 beats per minute was observed. There were no respiratory problems noted in the patient. Palpation revealed the abdomen to be smooth and painless. The bladder and bowel functions were normal. Upon assessment, the patient moved on their own and had a left lower limb limp. Examining the left thigh’s lateral side revealed a substantial, up to 20 cm long surgical scar that showed no symptoms of inflammation. In the area of the left hip joint, there was local moderate oedema, hyperemia of the tissues, and hyperthermia on palpation. The volume of movements of the left hip joint was significantly limited and painful. The initial laboratory investigations were normal. Daily activity, according to the Barthel index, score was 60, and the Karnovsky score was 40. Primary laboratory testing did not detect any pathology. Instrumental data showed no anomalies on the X-ray, but the electrocardiogram showed an AV conduction disturbance. On the sixth day of in-hospital stay, the patient had surgery—revision of endoprosthetic replacement with an antibiotic-loaded cement spacer. The patient experienced persistent hypotension in the postoperative period and required dopamine vasopressor support. Also, the patient developed posthemorrhagic anemia requiring hemotransfusions. The patient received complex therapy, including the continuous infusion of meropenem. Therapy included hemostatics in the form of sodium ethamsylate at 250 mg intravenously once in the postoperative period, narcotic and non-narcotic analgesics—morphine hydrochloride at 10 mg intravenously, promedol at 20 mg and ketoprofen at 100 mg intramuscularly. Gastroprotective therapy included omeprazole at 20 mg intravenously; the prophylaxis of thromboembolic complications involved taking enoxiparin at 0.4 mL subcutaneously, antiemetics—metoclopramide, antiarthymic treatment—and bisoprolol at 5 mg orally. Before starting continuous meropenem infusion, she received ceftriaxone at a dosage of 1000 mg twice daily.

**Figure 2:**
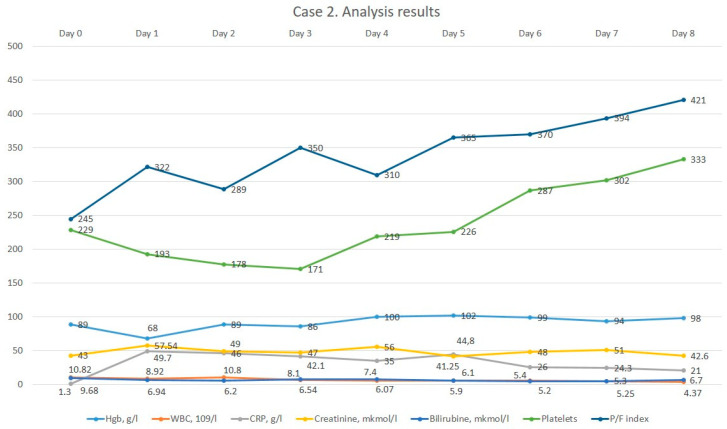
The dynamics of the 2nd patient’s analysis results.

The dynamics of laboratory results after the start of meropenem are shown in Figure 2. The patient was in intensive care for 4 days. Then, after the stabilization of her condition, she was transferred with improvement to a specialized department. The patient’s motor function had improved at the time of discharge; the Karnovsky index increased to 60, and Bartel index score increased to 80.

### 3.3. Case 3

A 63-year-old female patient was admitted with complaints of pain in the left lower extremity, restricted mobility, swelling of the leg, pain in the lumbar region, inability to move, increased body temperature up to 38 °C, chills, and general weakness. In 2008, she had a total endoprosthetic replacement of the left knee. Due to increasing pain syndrome and intoxication, she called an ambulance. The patient was admitted as an emergency to the clinic for septic shock, acute renal damage, electrolyte imbalance, and intoxication. During the time of admission to the hospital, taking into account complete anuria, a permanent catheter was urgently placed, and hemodialysis was performed. The electrolyte imbalance was corrected. On the background of infusion and diuretic, anti-inflammatory and antimicrobial therapy, the patient in dynamics went into polyuria (up to 4 liters per day). A decrease in azotemia (urea from 28 to 13 mmol/L, creatinine from 400 to 167 mmol/L) was noted. According to laboratory data, systemic inflammatory syndrome was detected (procalcitonin 13, CRP 170 units/L). Taking into account the laboratory data, bacterial blood culture and blood for sterility were taken from two veins, and ABT therapy (vancogen and amoxiclav) was selected on the recommendation of a clinical pharmacologist with a positive effect in reducing procalcitonin to 0.680, CRP 123; however, intoxication syndrome and pain in the knee joint, and restriction of mobility persisted. Taking into account these complaints, history of the disease, objective status, and laboratory instrumental data, periprosthetic infection could not be excluded. It was recommended to perform a puncture of the knee joint and to take a culture (as the blood showed Staphylococcus aureus). During puncture, purulent content was obtained. The patient was transferred to NSCTO, named after Batpenov, where further treatment was continued. Among the surgeries were a cesarean delivery, lumbar spine surgery in 2014, thrombosis of the right lower limb in May 2022, Cava filter implantation, and filter removal in November 2022. The patient’s background was complicated by the presence of rheumatoid arthritis for more than 20 years, for which she regularly took metipred at 4 mg. In addition, she suffered from arterial hypertension and had a myocardial infarction in August 2022, for which she underwent coronary stenting.

**Figure 3:**
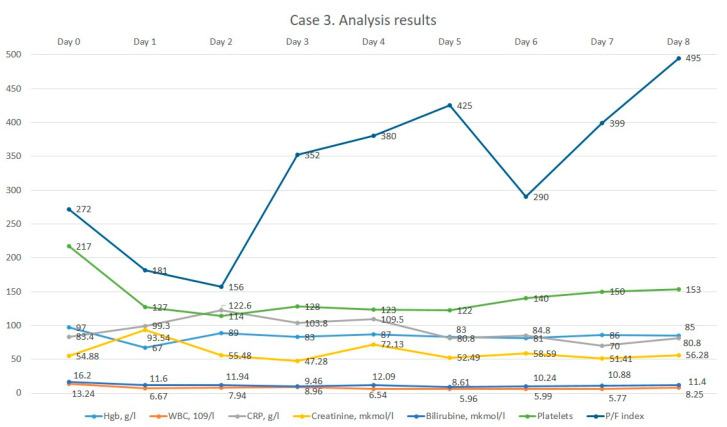
The dynamics of the 3rd patient’s analysis results.

Her general condition on admission was severe due to septic shock, hypotension, intoxication and acute kidney injury. Consciousness was clear, spoken speech was preserved, and no focal neurological or meningeal symptoms were detected. The position in bed was forced and sluggish with contact. Body temperature at admission was 37.0 °C. Skin and visible mucous membranes: clean, pale color. Bone and joint system: linear deviation of hands on both sides, swelling and soreness of large joints. Respiratory organs: breathing through the nose freely. Respiratory rate 20 per min chest: a regular shape, symmetrical. Vesicular breathing in the lungs: no rales. Cardiovascular system: heart tones muffled, rhythmic: HR 80 per min. Pulse 80 per min. There was no pulse deficit. Hemodynamics inclined to hypotension: BP 80/60 mmHg. Digestive system: tongue dry, covered with yellow plaque. The abdomen, on palpation, was soft and painless. Gases were expelled. Peristalsis was audible. The stool was the day before. Symptom of rubbing: negative on both sides. Urination: anuria by catheter. Swelling to the n/a of the tibia and hyperemia of the left lower limb, pronounced soreness on rotation and pastosity of the right lower limb. Status localis: the patient was in a forced supine position. Contours of the left knee joint were smoothed due to periarticular oedema. Palpation revealed sharp pain on the lateral surface of the left knee joint. Movement in the left knee joint was limited and painful. Local hyperemia and hyperthermia were determined. There were no neurovascular disorders in the periphery of the lower extremities. Wrist joints: ankylosis. Elbow joints showed persistent extensor contracture. Shoulder joints were limited in movement, sore and more on the left. Ulnar deviation of hands on both sides: synovitis of II, III MFS on the left and II finger on the right, painful on palpation. The hand could not fully clench into a fist. Strength in the hands was reduced. Knee joints asymmetrical with endoprosthesis in PCC, LKS painless and movements limited. Ankle joints were limited in movement and swollen. Morning stiffness before lunch: digital capillaritis. No enthesopathy and spinal stiffness. On admission, the patient’s consciousness was clear, speech was preserved and no focal neurological and meningeal symptoms were detected. The body temperature at admission was 37.0 °C. Respiratory system without pathology. From the side of the cardiovascular system, there were observed disorders in the form of instability of hemodynamics BP 80/60 mm Hg and inotropic support was connected. Urination through a urethral catheter; urination was normal. The patient was in a forced supine position. The contours of the left knee joint were smoothed due to periarticular oedema. Palpation revealed sharp pain on the lateral surface of the left knee joint. Movement in the left knee joint was limited and painful. Ankylosis of the wrist joints. Elbow deviation of the hands on both sides with synovitis of the fingers on the right side. On admission, the patient had baseline moderate anemia, leukocytosis and elevated CRP levels. According to the decision of the consilium, and taking into account the previous antibiotic therapy, it was decided to prescribe meropenem therapy. After obtaining the patient’s informed consent, continuous infusion of meropenem was started. The dynamics of the results of laboratory analyses are illustrated in Figure 3.

After the administration of meropenem therapy, a decrease in leucocytosis was noted, and CRP levels remained constant. On day 41 of hospitalization, the patient was transferred to the ward with improvement but was again transferred to the ICU on day 50 with anuria. The patient received comprehensive treatment, including vasopressor support, hemodialysis sessions, machine ventilation, antimicrobial therapy according to sensitivity, antifungal treatment, hemotransfusion, analgesia and hemostatic therapy. However, despite all the efforts, on the 105th day of hospitalization, the patient’s death was registered.

## 4. Results

During the study period, continuous infusion of meropenem was performed on three geriatric patients (Table 1). The median patient age was 65 years (Range 64–66), and 3/3 of patients were female.

As presented in Table 1, the mean length of ICU stay was 36 days (range 4–96 days), and the average length of stay in the hospital was 47 days (range 17–105 days). Two patients survived to hospital discharge, and one patient died on the 105th day of hospitalization. This patient was found to have resistant pseudomonas aeruginosa 10^4^.

Microbiological cultures were carried out before the start of continuous infusion, after 48 h, on days 3, 8 and 28 of the study.

In total, two elderly patients with periprosthetic joint infection-associated sepsis survived.

## 5. Discussion

The prevalence of elderly patients with severe sepsis and septic shock continues to grow rapidly worldwide. And in the near future, this may lead to a significant increase in the demand for ICU bed days among the elderly population as older patients have a longer length of stay in the ICU compared to younger people due to the presence of comorbidities and age-related changes in immune responses [6].

In this study, we applied the method of the continuous infusion of meropenem in the treatment of sepsis in geriatric patients (>60 years) with trauma in order to assess the clinical course of sepsis in these patients, as well as to evaluate clinical outcomes.

Prescribing antibiotics in elderly patients can be challenging. A proportional increase in body fat relative to skeletal muscle in the elderly may lead to an increase in the volume of distribution. With age, there is a decrease in the total size and number of nephrons, tubulointerstitial changes, thickening of the glomerular basement membrane, and intensification of glomerulosclerosis. This age-related histological appearance is often described as nephrosclerosis. Decreased clearance of the drug may be the result of a natural decline in renal function with age, even in the absence of renal insufficiency. Reduced clearance prolongs the half-life of drugs and leads to increased plasma concentrations of drugs in the elderly [7].

A decrease in the detoxification function of the liver contributes to the development of a pro-inflammatory condition, in which weakness may develop.

Because inflammation also suppresses drug metabolism, drugs given to frail older adults according to disease-specific guidelines may be subject to decreased systemic clearance, leading to adverse drug reactions, the further deterioration of function, and increased polypharmacy, exacerbating rather than improving the state of frailty [8].

It has long been recognized that beta-lactam antibiotics show a time-dependent effect on bacterial eradication. Long-term infusions of beta-lactam antibiotics achieve the target pharmacodynamic efficacy more effectively than short infusions. Thus, a prolonged infusion strategy may improve microbiological and clinical cures, especially when pathogens exhibit higher minimum inhibitory concentrations (MICs) [9,10]. But studies suggest that long-term or continuous infusions of carbapenems are associated with similar mortality rates as conventional intermittent infusions but may have other benefits, such as clinical cure and microbiological success, as evidenced by limited data [11,12,13].

In a single-center, randomized, open-label study in 240 adult ICU patients, the administration of meropenem as a continuous infusion resulted in similar mortality (16 percent) and clinical cure rates that were not statistically different (83 vs. 75 percent) compared to intermittent infusion, but rates of microbiological success (90% vs. 78%) were higher and ICU stay and duration of therapy were shorter with continuous infusion [14].

In a meta-analysis involving 632 patients with severe sepsis, the authors concluded that the continuous administration of beta-lactam antibiotics was associated with a reduction in hospital mortality [15]. But Joel M Dulhunty et al., in a multi-center study of 432 patients with sepsis with a mean age of 64 years, stated that there was no difference in outcomes between β-lactam antibiotic administration by continuous and intermittent infusion [16]. Perhaps these results are related to the average age of participants. After all, age is known to be an independent predictor of mortality in patients with severe sepsis [17,18].

In this study, we describe our experience of using meropenem in a continuous infusion in geriatric trauma patients with sepsis. This is because several population pharmacokinetics studies of meropenem in adults indicate that meropenem must be administered by a prolonged infusion in order to achieve microbiological success and subsequent clinical cure [19,20].

Population pharmacokinetic studies of meropenem in elderly patients are few, and research is mainly focused on investigating the optimal dosage regimen of meropenem. Thus, in China, there are 284 measurements of meropenem serum concentrations in 75 patients (aged 63–95 years). The CLCR and the APACHE II score have a significant effect on the pharmacokinetics of meropenem. In patients with lower respiratory tract infections (LRTI), a cut-off value of 76% for %T > MIC can be used to optimize the meropenem dosing regimen for clinical success [21]. In another prospective single-center open-label randomized controlled trial with 79 elderly patients with an LRTI, the authors concluded that the strategy for meropenem dosing based on a population PK/PD model can improve clinical response and avoid overtreatment in elderly patients with an LRTI [22]. But both PK studies of the optimal dosing regimen for meropenem were conducted using an intermittent infusion.

Only in one PK study with the participation of 178 elderly patients was the continuous infusion of meropenem used. Usman M, Frey OR and Hempel G concluded that an extended infusion of 1000 mg q8h can be considered for the empirical treatment of infections in elderly patients when CLCR is ≤ 50 mL/min. A continuous infusion of 3000 mg of the daily dose is preferred if CLCR > 50 mL/min. However, a higher daily dose of meropenem could be required for resistant strains (MIC > 8 mg/L) of bacteria if CLCR is >100 mL/min [23].

In the first few days, elderly patients have an improvement in the respiratory system after the application of continuous mode meropenem infusion, possibly due to an increase in the alveolar concentration of meropenem, but in some patients, this effect was short-lived. These patients subsequently required the intensification of antibiotic therapy. Studies show that the administration of meropenem by continuous infusion maintains higher concentrations in the subcutaneous tissue and plasma and, thus, better penetrates the lung tissue compared with intermittent bolus administration [24,25]. For instance, the PROMESSE study performed in 55 critically ill patients with severe pneumonia treated with 1 g/8 h reported a statistically higher AUC penetration ratio in the extended infusion group (3 h) compared to the intermittent group (mean (SD) 29 (±3) % vs. 20 (±3) % (*p* = 0.047) [26].

In a recently published MERCY randomized clinical trial with the participation of 607 individuals, the authors concluded that the continuous administration of meropenem did not improve the composite outcome of mortality and the emergence of pan-drug-resistant or extensively drug-resistant bacteria by day 28 [27].

In a systematic review with the meta-analysis of randomized trials comparing the short and prolonged infusion of beta-lactams, the authors recommended providing further studies in specific subgroups of patients according to age, sepsis severity, degree of renal dysfunction and immunocompetence [28].

Prolonged beta-lactam infusions have both advantages and disadvantages. The disadvantages of the prolonged method of infusion include the presence of constant intravenous access, the compatibility of the drug with other administered drugs, and the issue of the stability of the prepared solution. The instruction for branded meropenem (Merrem) notes that meropenem prepared for infusion in normal saline is stable for 1 h at room temperature and up to 15 h refrigerated. For meropenem (Santo, Shymkent, Kazakhstan) used in patients, no such information is available.

Using the continuous infusion of meropenem in geriatric patients with sepsis and periprosthetic infection, we noted a general trend in the form of improvement in the clinical picture of the patient. Specifically, the improvement of respiratory parameters is shown in the presented graphs in the form of an increase in the oxygenation index. This advantage of using meropenem reduces the days spent in the ICU. It has the potential for further research in this area, especially as the stock of available antibiotics is limited, and it is necessary to make the best use of available reserves. Since we encountered the emergence of a multidrug-resistant strain in the third case report, the question of the emergence of resistant strains using this infusion technique remains open.

Therefore, after analyzing the usage of the continuous infusion of meropenem in three geriatric patients, we concluded that the continuous infusion mode can probably lead to clinical improvement in the respiratory status of patients. Since our study has the following limitations in the form of a lack of therapeutic drug monitoring, we focus only on the clinical picture of patients. Further development of strategies for optimal dosing of meropenem and administration by continuous infusion in geriatric patients has the potential to increase clinical cure rates and decrease the demand for ICU beds.

## 6. Conclusions

In this article, we present the use of continuous meropenem infusion in geriatric patients with periprosthetic infection. The use of continuous meropenem infusion in the complex treatment of purulent-septic complications probably led to clinical improvement in this study. However, the emergence of new pan-resistant strains and overall mortality using this infusion technique remains unclear. Further high-quality randomized controlled trials in the elderly are needed.

## Figures and Tables

**Table 1:** Primary and secondary endpoints.

№	Age	Race	Comorbidity	Height	Weight	28-Day Survival	New Pan- Resistant Bacteria	Crops	Overall Mortality	Days in ICU	In-Hospital Days
1	66	Asian	Chronic ischemia of the brain of mixed genesis (hypertensive, atherosclerotic, vascular). Persistent residual effects of a stroke (from 2018). Left-sided spastic hemiplegia. Arterial hypertension. Iron deficient anemia	164	60	Alive	No	Pseudomonas aeruginosa 10^5^	Alive	9	20
2	65	European	Arterial hypertension. Atrial fibrillation and paroxysmal form.	166	69	Alive	No	Staphylococcus epidermidis 10^4^	Alive	4	17
3	64	Asian	Rheumatoid arthritis, chronic bronchitis and ischemic heart disease. Condition after myocardial infarction. Condition after stenting of the coronary arteries. Arterial hypertension.	152	71	Alive	Yes	Staphylococcus epidermidis 10^4^ Multi-resistant pseudomonas aeruginosa 10^4^	Death on 105th day	105	96

## Data Availability

The data presented in this study are available on request from the corresponding author. The data are not publicly available to ensure the confidentiality of the patients’ personal information.
